# Effectiveness guidance document (EGD) for Chinese medicine trials: a consensus document

**DOI:** 10.1186/1745-6215-15-169

**Published:** 2014-05-13

**Authors:** Claudia M Witt, Mikel Aickin, Daniel Cherkin, Chun Tao Che, Charles Elder, Andrew Flower, Richard Hammerschlag, Jian-Ping Liu, Lixing Lao, Steve Phurrough, Cheryl Ritenbaugh, Lee Hullender Rubin, Rosa Schnyer, Peter M Wayne, Shelly Rafferty Withers, Bian Zhao-Xiang, Jeanette Young, Brian M Berman

**Affiliations:** 1 Institute for Complementary and Integrative Medicine, University Hospital Zurich, Zurich, Switzerland; 2 Center for Integrative Medicine, University of Maryland, School of Medicine, Baltimore, MD, USA; 3 Department of Family and Community Medicine, University of Arizona, Tucson, AZ, USA; 4 Group Health Research Institute, Seattle, WA, USA; 5 Department of Medicinal Chemistry and Pharmacognosy, University of Illinois at Chicago, Chicago, IL, USA; 6 Kaiser Permanente Northwest, Center for Health Research, Portland, OR, USA; 7 Complementary and Integrated Medicine Research Unit, Department of Primary Care, University of Southampton, Southampton, UK; 8 Research Department, Oregon College of Oriental Medicine, Portland, Oregon, USA; 9 Center for Evidence-Based Chinese Medicine, Beijing University of Chinese Medicine, Beijing, China; 10 Complementary Medicine Program and Integrative Medicine, University of Maryland Medical Center, Baltimore, MD, USA; 11 Centers for Medicare and Medicaid Services, Baltimore, Maryland, USA; 12 School of Nursing, The University of Texas at Austin, Austin, TX, USA; 13 Osher Center for Integrative Medicine, Division of Preventive Medicine, Brigham and Women’s Hospital and Harvard Medical School, Boston, MA, USA; 14 The Institute for Integrative Health, Baltimore, MD, USA; 15 School of Chinese Medicine, Hong Kong Baptist University, Kowloon Tong, China; 16 Patient stakeholder, New York, NY 10001, USA

**Keywords:** Comparative effectiveness research, Effectiveness guidance document, Chinese medicine research

## Abstract

**Background:**

There is a need for more Comparative Effectiveness Research (CER) on Chinese medicine (CM) to inform clinical and policy decision-making. This document aims to provide consensus advice for the design of CER trials on CM for researchers. It broadly aims to ensure more adequate design and optimal use of resources in generating evidence for CM to inform stakeholder decision-making.

**Methods:**

The Effectiveness Guidance Document (EGD) development was based on multiple consensus procedures (survey, written Delphi rounds, interactive consensus workshop, international expert review). To balance aspects of internal and external validity, multiple stakeholders, including patients, clinicians, researchers and payers were involved in creating this document.

**Results:**

Recommendations were developed for “using available data” and “future clinical studies”. The recommendations for future trials focus on randomized trials and cover the following areas: designing CER studies, treatments, expertise and setting, outcomes, study design and statistical analyses, economic evaluation, and publication.

**Conclusion:**

The present EGD provides the first systematic methodological guidance for future CER trials on CM and can be applied to single or multi-component treatments. While CONSORT statements provide guidelines for reporting studies, EGDs provide recommendations for the design of future studies and can contribute to a more strategic use of limited research resources, as well as greater consistency in trial design.

## Background

Chinese medicine (CM) includes a broad range of medical practices that have many of their roots in China and share common theoretical concepts. According to the description by the National Center for Complementary and Alternative Medicine (NCCAM) CM ‘encompasses many different practices, including acupuncture, moxibustion (burning an herb above the skin to apply heat to acupuncture points), Chinese herbal medicine, *tuina* (Chinese therapeutic massage), dietary therapy, and *tai chi* and *qigong* (practices that combine specific movements or postures, coordinated breathing, and mental focus) [1].

In general, CM follows a theoretical framework, and the etiology and pathogenesis of CM uses its own terminology. The processes of diagnoses and interventions of this medical system are different from those in conventional medicine, and both are guided by traditional principles of CM. CM is often used as a multi-component treatment in which cultural, philosophical, historical, temporal, and geographic aspects as well practitioner training, all influence its heterogeneity.

From the practitioner’s perspective, CM diagnoses *(*for example, *bian zheng),* often also called CM patterns or CM syndromes differentiation, inform CM interventions. To date, treatment individualization according to the CM diagnoses seems to have very little clinically relevant impact on the outcome of acupuncture treatment in clinical studies [2-4], whereas it might be more relevant for clinical trials of CM pharmacotherapy [5,6], although evidence is still scarce. In practice, CM treatment is often individualized, and because CM diagnoses may change over time, interventions can also change during the course of treatment. Currently in China, standardization of CM diagnoses and treatment for practice and research is emphasized, whereas in the West, a trend toward more individualization in research protocols and in practice is observed.

### Aim of the document

This document provides consensus advice for the design of comparative effectiveness research (CER) trials in CM for researchers. CER is the generation and synthesis of evidence that compares the benefits and harms of alternative treatment options to prevent, diagnose, treat, and monitor a clinical condition or to improve the delivery of care. The purpose of CER is to assist consumers, clinicians, purchasers, and policy makers to make informed decisions that will improve health care at both the individual and population levels [7].

CER broadly aims to ensure more adequate design and optimal use of resources in generating evidence for CM to inform stakeholder decision-making. These consensus recommendations can be applied to single and multi-component interventions. They are based on the assumption that a reduction of internal validity can be justified in order to increase authenticity of the intervention and setting, thereby enhancing generalizability, relevance, feasibility and timeliness of research results.

## Methods

The development of the Effectiveness Guidance Document (EGD) followed a structured and predefined consensus process, which included a pre-workshop online survey (April 2012), a consensus workshop (19 May 2012 in Portland, OR, USA), and three written Delphi rounds (August 2012, January 2013 and May 2013) utilizing written comments to finalize the document.

Multiple stakeholders were involved in the consensus process for this EGD to balance aspects of internal and external validity in the recommendations. Participants of the workshop had the following backgrounds: one CM patient, one health insurance representative, nine experts in CM with experience in both CM practice and CM research (two from China, two with a Chinese background living in the USA, four from the USA, and one from UK), and 6 methodologists (with backgrounds in clinical research, statistics or epidemiology, 5 of them with experience in CM research). The consensus meeting utilized presentations, large group discussions and an adapted *world café* methodology. The world café method, as developed by Brown and Isaac, is a simple, effective, and flexible format for facilitating large group dialogue [8]. It has been used in the development of prior EGDs [9] to foster collaborative dialogue, knowledge sharing, and community participation in a setting that involves multiple stakeholder groups.

Expert involvement was further broadened by the inclusion of nine international CM research experts who did not participate in the workshop, but who contributed to the survey and both Delphi rounds. The consensus process was finalized after feedback from all workshop participants and the external review experts.

## Results

The results of the consensus process are presented in two sections: I) Using available data, II) recommendations for future clinical studies.

I) Using available data

1) Clinical Health Records

a) Data from clinical health records are important and can be very useful for generating hypotheses for future randomized trials, identifying common diagnostic patterns and promising interventions, assisting recruitment for prospective studies, correlating CM diagnoses with outcomes, and providing information on patient characteristics and interventions in usual care.

b) The potential for CER based on clinical health records that document CM use (for example, Kaiser Permanente California [10,11], Veterans’ Administration [12]) needs more exploration. The value of such records depends on the documentation structure and the availability of data on useful surrogate outcomes such as visits, days off from work, drug use, and/or costs.

2) Data from previous trials

a) Databases provide helpful information on existing CM trials (for example, Oregon College of Oriental Medicine for Acupuncture (AcuTrials**®**) [13], New England School of Acupuncture (NESA) Database [14], Chinese BioMedical Literature Database (CBM) [15], China Network Knowledge Infrastructure (CNKI) [16], and Chinese Scientific Journals Database (VIP) [17]).

b) There is a need to develop a database of CM trials that have included multi-component treatments.

c) Patient level raw data from randomized trials on CM should be shared in order to generate a database; this should be especially encouraged for all new trials. Guidelines to facilitate such sharing should be developed and implemented.

II) Recommendations for future clinical studies

The recommendations are summarized under the following headings:

● Designing CER studies

● Treatments, expertise and setting

● Outcomes

● Study design and statistical analyses

● Economic evaluation

● Publication

Design and reporting guidelines relevant to these recommendations are summarized in Table 1. A checklist highlighting the most important elements is presented in Table 2.

**Table 1:** Relevant guidelines for design and reporting

**Document**	**Design**	**Reporting**	**Reference**
Effectiveness guidance document for acupuncture research	X		[9]
SPIRIT for content of clinical trial protocols	X	X	[18]
CONSORT for parallel group randomized trials		X	[19]
CONSORT extension for pragmatic trials		X	[20]
CONSORT for non-pharmacological trials		X	[21]
CONSORT extension for cluster randomized trials		X	[22]
CONSORT extension for acupuncture trials		X	[23]
CONSORT extension for herbal interventions		X	[24]
CONSORT extension for non-pharmacological treatment interventions		X	[25]
CONSORT extension for traditional Chinese medicine		X	[26]
CONSORT extension for patient reported outcomes		X	[27]
Guidelines for randomized controlled trials investigating Chinese herbal medicine		X	[28]
Extending the CONSORT statement to moxibustion		X	[29]

CONSORT = Consolidated Standards of Reporting Trials.

**Table 2:** Checklist for the most relevant aspects of comparative effectiveness research for Chinese medicine clinical studies

**Designing comparative effectiveness research (CER) studie**	
1. Stakeholder involvement	All relevant stakeholders are involved in identification of research topic, plan and design of CER, interpretation of results
2. Efficacy-effectiveness continuum	Location on the efficacy-effectiveness continuum is determined for participant selection/eligibility criteria, treatment protocol, practitioner expertise, outcomes, and setting in which the study is conducted
3. Study design	Designs for multi-component interventions should be considered
**Study population**	
4. Eligibility criteria	Should be as broad as possible in the context of available resources - Study population includes both CM-naïve and CM-non- naïve patients
5. Diagnoses	Recruitment of patients should follow Western diagnoses - CM diagnoses should be done whenever possible
6. Patient recruitment	Patients are recruited from site(s) where the treatment is usually provided
**Treatment, expertise, and setting**	
7. Defining treatments	If intervention involves multi-component treatment the combination should be plausible and feasible in usual care - non-CM best practice alternatives are based on guidelines or broad expert consensus
8. Acupuncture	See [9]
9. Qi gong/tai chi	Style and setting should reflect typical community-based programs
10. Herbal medicine	Local and national regulations should be taken into account
11. Treatment documentation	Documentation reflects which treatments were received by all groups (interventions and co-interventions)
**Outcomes**	
12. Measures	Widely accepted or standardized outcome measure used - secondary outcomes capture relevant patient-centered dimensions for the condition under study
13. Timing	Assessment schedule is balanced allowing study to acquire relevant data without substantial disruption of treatment or setting
**Study design and statistical analysis**	
14. Allocation	Allocation is concealed - stratification for subgroups and/or dynamic allocation for key characteristics are used
15. Blinding	Outcome data are kept inaccessible to practitioners - blinded outcome rater is used if possible
16. Preferences/expectation	Preferences and expectations are measured at baseline
17. Sample size	Sample size takes patient heterogeneity into account - required sample size is feasible - Study has enough power for planned subgroup analyses
18. Subgroups	Relevant subgroups are pre-planned - exploratory subgroup analysis is mentioned in study aims
19. Statistical analysis	Intention-to-treat analyses are planned - relevant subgroup analyses are planned - data analyses are adjusted for stratification variables, baseline differences and relevant confounders
**Economic evaluations**	
20. Relevance	Setting reflects reality in clinical practice
21. Methodological approach	Standard methods for economic evaluations are used - sensitivity analysis is employed for all relevant stakeholder perspectives - relevant subgroups are identified
22. Observation time	Long-term observation (≥12 months) are planned if possible
**Publications**	
23. Guidelines	Relevant guidelines (CONSORT) are consulted and followed
24. Content	Statements of how and why this is CER are included - study setting (including practitioner selection procedure) is described in detail - treatment group description from informed consent is provided - comparison groups are described in detail - data are provided on all interventions and co-interventions received - relevant subgroup analyses are reported

CM, Chinese medicine; CONSORT, Consolidated Standards of Reporting Trials.

### Designing CER studies

1) Stakeholder engagement

a) Involvement of all relevant stakeholders (for example, practitioners, clinicians, patients, payers, researchers) is highly relevant for CER when identifying research questions, planning, and designing the study, and interpreting study results.

b) Stakeholder involvement should follow a systematic approach (for more information on suitable methods see Deverka et. al. [30]).

2) Study question and the efficacy-effectiveness continuum

a) The study question should be clearly phrased, and include all relevant information about study participants, interventions, comparison groups and outcome parameters. In particular, it should clarify whether the CM treatment is to be assessed as an “alternative” in direct comparison, using a superiority or non-inferiority hypothesis, or as an adjunct to a usual or standard care treatment.

b) During the trial planning phase, time should be given to discuss and determine the trial’s position along the efficacy-effectiveness continuum [31]. Use of the PRECIS tool to support this process is recommended [32].

3) Study designs for complex multi-component CM interventions

a) Pragmatic trials may be used to compare complex multi-component treatment alternatives (for example, multi-component CM treatment, consisting of acupuncture, dietary advice and *qigong* compared to multi-component conventional treatment, consisting of prescribed recommendations for exercise and diet for the treatment of mild hypertension) [20].

b) Multi-arm trials may help to identify dosing effects, synergistic effects when combining different CM interventions, and effective components within one treatment modality (for example, isolating meditative and breathing components from comprehensive *qigong*/*tai chi* protocols).

c) The complexity of therapeutic decision-making and treatment changes within the treatment process could be reflected by designs as demonstrated by Ritenbaugh et. al. [33,34].

### Study population

4) General eligibility criteria

a) In the context of available resources, eligibility criteria should be as broad as possible. The criteria should reflect the evidence of the pattern of usage and disease burden, and the study population should reflect all well-known relevant disease characteristics that may interact with the treatment (for example, gender, disease stage, comorbidities, co-medications).

b) Patients with comorbidities should not be explicitly excluded from the study enrollment unless the comorbidities make them inappropriate candidates for the treatment, but safety and regulatory aspects have to be taken into account (for example, when using herbs).

c) Both CM naïve and non-naïve patients should generally be considered eligible for study inclusion to reflect real-world patient population. If special groups are targeted, the rationale should be provided.

5) Diagnosis

a) The study disease/condition should be defined as clearly as possible from the Western medical approach as well the CM approach.

b) In general, recruitment of patients should initially follow the Western diagnostic approach.

c) CM diagnoses should subsequently be made in all treatment groups (before randomization in randomized studies) and documented whenever possible. A clear, comprehensive and well recognized categorization system for the CM diagnoses should be used. The CM diagnoses could be used for exploratory analyses and to generate hypotheses for future studies (for example, impact on the outcome, correlation with genomics, prediction of the course of disease and response to treatment in the different treatment groups).

d) Designing the study around CM diagnoses in general should be avoided (for example, including only patients with one type of CM diagnosis), because results are difficult to implement into a medical system that follows a Western approach. However, if a clear and limited number of CM diagnoses exist, and research-based evidence is available to suggest that the CM diagnoses are reliable, consistent across practitioners, and have a relevant influence on the intervention or the outcome, this should be taken into account, (for example, by stratified randomization according to CM diagnoses, or different treatments for different CM diagnoses).

e) If the CM diagnosis is relevant to the study design (for example, preplanned confirmatory subgroup analyses according to the CM diagnoses), emphasis should be placed on strengthening its reliability (for example, assessing inter-rater agreement, providing training and/or calibration of practitioners).

6) Patient recruitment

a) Recruitment should be carried out systematically and from various sources (for example, in places where the population suffering from the disease is available and/or relevant treatments are usually employed).

b) Patients’ treatment should be recorded at baseline and efforts should be made to recruit both those who express treatment preferences and those who do not.

c) Appropriate strategies to ensure successful recruitment should be developed and, if possible, pretested.

d) In non-randomized studies, as far as possible, recruitment strategies should be similar for all treatment arms.

### Treatment, expertise and setting

7) Defining treatment groups

a) The treatment alternatives (CM treatments and non-CM treatments) should each provide value to the patient by having the potential to be “best practice” [7]. In the absence of a clear evidence base “best practice” of CM can be derived by 1) reviewing alternatives that have been effective in addressing similar issues in the past and could be applied to a current problem, and 2) integrating information from a number of sources (recommended research protocols, existing clinical data, reference to classical usage, and formal consensus procedures). If a direct “head-to-head” comparison is used, all treatment options should reflect usual care as much as possible, and ideally the extent of standardization should be similar in all treatment groups. If CM treatment plus usual care is compared to usual care alone, usual care should be similarly defined and provided in both groups.

b) The comparison treatments and their complexity should have widespread clinical acceptance relative to the condition studied and stage of disease.

c) The rationale for the CM treatment should follow one of the following two approaches:

○ If there is positive evidence for single treatment components from clinical studies, these components may be combined to evaluate the comparative effectiveness of a complex multi-component treatment. The combination of treatment components should be plausible and feasible in a routine care context and not contradicted by CM theory.

○ If convincing clinical evidence is not available for the single treatment components, overall effectiveness of a multi-component CM treatment should be evaluated before component efficacy is addressed. The rationale for the treatment components and their combination should be based on both thorough clinical evidence and associated theory from the literature.

d.) Non-CM comparator treatments should be based on evidence, guidelines or broad expert consensus.

8) Special aspects for acupuncture have been described in the EGD for acupuncture research [9].

9) Special aspects of *qigong/tai chi*

a) A large number of Qigong/Tai Chi styles exist and the chosen style(s) for a research trial should represent to some extent the real world heterogeneity of practice in the country where the study is performed.

b) If a very specific style is used, it must be justified (specific style or protocol found to be effective in prior studies, or potential for widespread adoption), and limits to generalizability should be acknowledged.

c) The setting in which *qigong* and *tai chi* is offered should be accessible and reflect typical community-based programs; the longer-term (post-study) sustainability of the program should be considered (access to training, classes or instruction).

d) *qigong*/*tai chi* should be provided by qualified instructors, with expertise in both protocol content and teaching. Training and teaching qualifications of all instructors should be reported. In studies of high-risk populations, treatment safety should play a prominent role, using more expert instructors and protocols validated with respect to safety.

e) Studies should provide information that specifies exercises (names and style), dose (number and duration of classes, home exercise) and ancillary training materials offered (for example, books and audio-visual material).

10) Special aspects of herbal medicine

a) Study designs must comply with local and national regulations regarding herbal medicines in the country of the clinical trial. Although widely used in everyday practice, and in spite of the fact that research is urgently needed, research on individualized, multi-herb formulations is very difficult to accomplish in most Western countries due to government regulations and Institutional Review Board approval.

b) The treatment should be based on existing evidence (systematic review of Chinese as well as European and US-based databases, survey of normal practice, practitioner case records, etc.), should have “model validity” within CM, and provide a rationale for “good practice” in CM.

c) It must be assured that the treatment does not include any endangered species.

d) Herbal formulations that include different herbs need to be adequately defined (chemically to assure quality of herbs).

e) Additional safety aspects have to be taken into account including the aspect that relevant laboratory tests should be performed during the trial to check for potential unwanted side effects interactions with other medicines, although the latter can impact study design, cost and recruitment.

f) Relevant aspects for the different stakeholder groups should be taken into account when planning the treatment. This includes consideration of:

○ Different preferences among different stakeholder groups for the route of administration (for example, decoction, granules, or tablets). However, when making changes to improve participant compliance, care should be taken to maintain treatment regimes and dosages that agree with standards of “good practice”.

○ Costs of the treatment, for the research trial per se and for the likelihood of trial results informing clinical practice. Cost may be affected by the complexity of the herbal formula, as well as the dosage and route of administration. These are important considerations for payers and patients who often pay out-of-pocket.

11) Treatment documentation

a) All treatments (study treatment, co-treatments, over-the-counter self-medication) carried out in all groups should be documented as well as the context in which the usual care is provided. A variety of documentation methods and sources may contribute details (including medical records, case report forms, patient diaries).

### Outcomes

12) Measures

a) The main outcome measures should be patient-centered and, if appropriate, include relevant biological measures for the respective disease/condition. Whenever possible, diagnosis-specific validated standards for outcome measures published by professional associations (for example, the International Headache Society on headache measures) [35] or broad published expert consensus (for example, on back pain measures) [36] should be followed to permit better comparison of study results.

b) If no standard measures for targeted outcomes exist, or those that are available are not suitable for CER on CM, new outcome measures should be developed. Stakeholders should be involved in this process and qualitative and quantitative research methods should be used in combination.

c) Multiple primary outcomes addressing distinct dimensions may be used, if appropriate. The use of multiple primary outcomes should be addressed in the sample size calculation and statistical analysis plan.

d) Secondary outcome measures should capture relevant patient-centered dimensions of the respective disease/condition (both self-reported and biological, as well as outcomes relevant within the CM model for the treated CM diagnoses). Additional secondary outcomes might, if appropriate, include measures of collateral effects (i.e., positive and negative consequences of the treatment experience, often seemingly unrelated to the main outcomes).

13) Timing

a) Outcomes should be evaluated over a sufficient long period to capture true impact on chronicity of disease and to distinguish this from short term or intermittent relief.

b) The use of multiple intervals to document and compare the trajectory and persistence of treatment effects is recommended. Data collection methods that do not have a direct influence on the treatment plan (for example, text messages, phone calls, smart phone applications) are recommended. However, the frequency of assessment should be balanced, so that relevant information is gained without major disruptions of treatment implementations or practice setting and with minimal risk of respondent overload.

### Study design and statistical analysis

14) Allocation methods

a) Use of appropriate allocation methods is strongly recommended. Randomization at the level of individual patients is still the most frequently used method, but dynamic allocation procedures (for example, rank minimization) may be used as an alternative. The final choice depends on the design of the study and the sites at which the study will be conducted [37].

b) Stratified randomization or adaptive allocation techniques may be used to prevent imbalances for relevant covariates and potential confounders in study arms [38,39].

c) Partially randomized patient preference designs have an advantage in that they provide additional exploratory information as to whether the results observed for randomized patients are different from those who were not randomized because of treatment preferences. However, these designs, while adding potentially important outcome data to a clinical trial, are often not feasible because of the need for much larger sample sizes and higher costs [40].

d) Cluster randomization is the best approach under circumstances where the randomization of social units (for example, clinics) is advisable to avoid contamination of treatments between groups. When planning such a trial, it is necessary to consult the relevant literature and local institutional roles to determine from whom, when, and how informed consent must be obtained [41], and to take into account that a larger sample might be needed than in patient level randomized trials [42], because the trials are powered based on the number of participating units.

e) Standard procedures ensuring allocation concealment (for example, central randomization or secure databases) should be employed.

15) Blinding

a) Blinded outcome measurement (for example, a blinded rater) is recommended in order to reduce bias, especially for outcomes that, in usual clinical practice, are assessed by the practitioner (for example, physical assessments). Methods to minimize the risk of unblinding (for example, allocation concealment, rater training, standardized assessment protocol) should be employed.

b) Data analyses should be blinded whenever possible.

c) Outcomes data reported by patients for the study purpose (for example, quality of life assessment) should be kept inaccessible to the practitioner (for example, by using sealed envelopes or preferably by sending questionnaires directly to a study office independent of the study site or using a blinded interviewer).

d) Recommendations for blinding the treatment (for example, when using a double dummy placebo for the comparison of herbal medicine with conventional drugs) are provided in the guidelines developed in the European Union funded GP-TCM project [43].

16) Patient preferences and expectations

a) Patient preferences should, if appropriate, be acknowledged in the study design, e.g., by using a partially randomized patient preference design. If such a design is not feasible, then it is important to document both the patients’ preferences regarding the treatment options available in the trial as well as the degree of their knowledge and experience with these treatment options.

b) Assessing patient and practitioner preferences and expectations for the treatments offered in the study at baseline should be considered. In randomized trials they should be assessed before randomization and for all available treatment options.

17) Sample size

a) Sample size should focus on the main outcome(s) and the minimum clinically important difference (MCID) for the respective outcome(s) and take into account greater heterogeneity in CER study populations. Because of this, researchers should specifically avoid conducting small trials (< 50 patients per arm) in CER, unless there is a specific reason to do such studies (for example, pilot studies to test feasibility and recruitment).

18) Subgroups

a) Relevant subgroups for the disease/condition under study should be identified based on existing data and the literature. Also of current interest are subgroup analyses for different CM pattern diagnoses and for CM patients who are naïve/non-naïve to CM. If sample size permits, further analyses can be carried out for gender, age, ethnicity, disease severity/duration, treatment preference and recruitment site.

b) The main subgroup analyses should be pre-specified in the analysis plan and included in sample size planning for confirmatory testing. Further subgroup analyses can be done on an exploratory level, but should be stated as an objective in the study protocol.

19) Statistical analysis

a) Primary analysis for superiority trials of CM should be pre-specified and intention-to-treat. In order to assess real-world effectiveness of treatments, benefits and harms should be compared in relation to the treatment to which patients were assigned.

b) Analyses should adjust for relevant potential confounders (for example, baseline value of the outcome measure, stratification variables, expectation, and baseline CM diagnosis).

c) Especially in non-randomized studies, procedures to compensate for baseline differences must be used (for example, matching and/or adjusted analysis).

### Economic evaluations

20) Relevance

a) Comparing the effectiveness of treatment options should be the primary aim of CER, but economic evaluations should be included whenever possible as a secondary aim.

b) To allow realistic cost estimates, the setting(s) of the study should reflect the real-world clinical practice for each treatment as closely as possible. If a study includes a standardized and a non-standardized CM arm, it would be useful to compare their cost-effectiveness.

21) Methodological approach

a) Standard effectiveness measures for economic evaluations should be employed that include both benefits and harms (for example, utility measures based on SF-36, SF-12 or EQ-5D) [44].

b) Economic evaluations should be designed to reflect stakeholder perspectives with sensitivity analysis performed, whenever possible, from different stakeholder perspectives (for example, society, payer and patient). Because CM is often paid out-of-pocket, the patient’s perspective is highly relevant.

c) Requirements of the local context (for example, guidelines by regulatory agencies) should be taken into account.

d) Subgroup analyses should mainly focus on subgroups defined a priori for the effectiveness study. Additional analyses should be clearly described as exploratory. A subgroup analysis for gender is recommended since there is preliminary evidence that gender may influence the cost-effectiveness of CM treatment [45].

e) Exploratory analyses of factors that predict a better cost-effectiveness are suggested to develop future hypotheses.

22) Observation time

a) Long-term observations with intermediate measurement time points are highly recommended for economic evaluations of chronic disease in order to evaluate development of cost-effectiveness over time.

### Publication

23) Existing guidelines

To ensure that CER on CM will fulfill reporting standards, the relevant CONSORT guidelines should be followed (see Table 1).

24) Content

a) Publication of a detailed study protocol (design publication) should take place whenever possible prior to the recruitment of the last patient.

b) The study should be registered in an internationally accessible trial database with as many details as possible provided.

c) Publication of the completed study should describe why and how it qualifies as CER and make clear the phase of the study.

d) The setting of the study should be described, including information about the typical care setting in the country where the study was performed (and, if relevant, in other countries). The procedure for selection of practitioners for each treatment group should be described, with an account of whether and how those included in the study differ from the average practitioner (for example, training, experience).

e) Information on how patients were informed about the treatment options should be provided.

f) If a usual-care or standard-care comparison group is used, a detailed description with citations for standard care should be included in the intervention section.

g) Detailed results of all treatments should be presented; adherence to interventions and co-interventions should be reported for each group.

h) Whenever possible, the most relevant subgroup analyses and analyses of patient characteristics that predict a better outcome should be published together with primary results. Detailed subgroup analysis and/or de-identified patient level data can be provided as online files.

## Discussion

This is the first EGD for clinical research involving a complex and multi-component medical system, providing detailed advice for the design of CER in the field of Chinese medicine for single as well as multi-component treatments. This EGD has been derived from a systematic development process, with active involvement of different stakeholders from the West and China, and aims to inform researchers inside and outside China when designing their trials. The involvement of China-based stakeholders reflects both the geographic roots of CM and a growing interest in CER studies in China. During the development process, stakeholder groups uncovered a broader understanding of the complexity of a multi-component treatment, the cultural differences in CM practice and research between China and other countries, and the resulting challenges for the study design. The heterogeneity of CM as practiced in different countries made it necessary to develop recommendations that account for these variances of style and context. China has a strong research focus on herbal medicine, however this is less common in other countries due to regulatory requirements. Herbal medicine trials have unique challenges and within this consensus process we were only able to discuss the most prominent ones. For CM herbal medicine trials, it is recommended that the guidelines for randomized controlled trials investigating Chinese herbal medicine be utilized [28]. A limitation of consensus procedures is that not all aspects of the study design can be addressed. For example, no recommendations for the study sites were discussed.

There was discussion about the adequacy of comparison groups, but because of the broad range of optional research questions and the multifold combination of interventions, no detailed recommendations were made. However, there was consensus that the comparison group(s) should have the option for best practice and should be based on guidelines or broad expert consensus as recommended under point 7.

Within the process, several methodological aspects unique to CM were identified that need further research and clarification. For example, the CM diagnosis classification system and the heterogeneity of its application need research to ensure overall validity and reliability. Another example is that the CM treatment benefits should also be measurable in CM terms. This goal may be complicated by aspects of CM’s explanatory model that aims to restoring balance or increasing resilience, for which suitable outcome measures are not yet developed.

## Conclusion

Although CONSORT statements provide guidelines for reporting studies, EGDs provide recommendations for the design of future studies and can contribute to a more strategic use of limited research resources as well as greater consistency in trial design. In particular, the present EGD provides the first set of systematic methodological guidance for future CER on CM.

## Abbreviations

CER: comparative effectiveness research; CM: Chinese medicine; CONSORT: Consolidated Standards of Reporting Trials; EDG: Effectiveness Guidance Document; MCID: minimum clinically important difference; NCCAM: National Center for Complementary and Alternative Medicine.

## Competing interests

The workshop was funded by The Institute for Integrative Health (TIIH), Baltimore MD, USA, a non-profit organization. Brian Berman is the president of TIIH, which supported Claudia Witt with a travel grant for the submitted work.

## Authors’ contributions

CMW: designed the study, coordinated the consensus process, participated in the workshop and Delphi rounds, collected and analyzed the data, wrote the first draft of the work, revised the manuscript and approved the final version. MA: participated in the workshop and Delphi rounds, revised the work critically for important intellectual content and approved the final version, DC, CC, CE, AF, RH, JL, LL, SP, CR, RS, PW, BZ and BB: participated in the workshop and Delphi rounds, revised the work critically for important intellectual content and approved the final version, LR and JY: participated in the workshop and Delphi rounds and approved the final version, SW: participated in the coordination and data collection and the workshop, revised the work critically for important intellectual content and approved the final version, CMW and BB are guarantors for the paper and accept full responsibility for the work and controlled the decision to publish.
